# Comparative analysis of ACE2 protein expression in rodent, non-human primate, and human respiratory tract at baseline and after injury: A conundrum for COVID-19 pathogenesis

**DOI:** 10.1371/journal.pone.0247510

**Published:** 2021-02-24

**Authors:** Sourabh Soni, Yujie Jiang, Yohannes Tesfaigzi, Jason L. Hornick, Sule Çataltepe

**Affiliations:** 1 Department of Pediatric Newborn Medicine, Brigham and Women’s Hospital and Harvard Medical School, Boston, Massachusetts, United States of America; 2 Department of Respiratory Medicine, The Affiliated Hospital of Youjiang Medical University for Nationalities, Baise, Guangxi, China; 3 Division of Pulmonary and Critical Care Medicine, Department of Medicine, Brigham and Women’s Hospital and Harvard Medical School, Boston, Massachusetts, United States of America; 4 Department of Pathology, Brigham and Women’s Hospital and Harvard Medical School, Boston, Massachusetts, United States of America; University of Alabama at Birmingham, UNITED STATES

## Abstract

Angiotensin converting enzyme 2 (ACE2) is the putative functional receptor for severe acute respiratory syndrome coronavirus 2 (SARS-CoV-2). Current literature on the abundance and distribution of ACE2 protein in the human respiratory tract is controversial. We examined the effect of age and lung injury on ACE2 protein expression in rodent and non-human primate (NHP) models. We also examined ACE2 expression in human tissues with and without coronavirus disease 19 (COVID-19). ACE2 expression was detected at very low levels in preterm, but was absent in full-term and adult NHP lung homogenates. This pattern of ACE2 expression contrasted with that of transmembrane protease serine type 2 (TMPRSS2), which was significantly increased in full-term newborn and adult NHP lungs compared to preterm NHP lungs. ACE2 expression was not detected in NHP lungs with cigarette smoke-induced airway disease or bronchopulmonary dysplasia. Murine lungs lacked basal ACE2 immunoreactivity, but responded to hyperoxia, bacterial infection, and allergen exposure with new ACE2 expression in bronchial epithelial cells. In human specimens, robust ACE2 immunoreactivity was detected in ciliated epithelial cells in paranasal sinus specimens, while ACE2 expression was detected only in rare type 2 alveolar epithelial cells in control lungs. In autopsy specimens from patients with COVID-19 pneumonia, ACE2 was detected in rare ciliated epithelial and endothelial cells in the trachea, but not in the lung. There was robust expression of ACE2 expression in F344/N rat nasal mucosa and lung specimens, which authentically recapitulated the ACE2 expression pattern in human paranasal sinus specimens. Thus, ACE2 protein expression demonstrates a significant gradient between upper and lower respiratory tract in humans and is scarce in the lung. This pattern of ACE2 expression supports the notion of sinonasal epithelium being the main entry site for SARS-CoV-2 but raises further questions on the pathogenesis and cellular targets of SARS-CoV-2 in COVID-19 pneumonia.

## Introduction

Coronavirus disease 19 (COVID-19) pandemic, caused by the severe acute respiratory syndrome coronavirus 2 (SARS-CoV-2), has accounted for more than 90 million confirmed cases and close to 2 million deaths worldwide as of January 2021. The pathogenesis of COVID-19, which primarily targets the respiratory system, remains to be fully elucidated. The first and essential step in all coronavirus infections is the binding of the viral spike protein (S protein) to a receptor on host cells [1, 2]. Angiotensin converting enzyme 2 (ACE2), a type I transmembrane glycoprotein with carboxypeptidase activity, has been proposed to be the key functional receptor for SARS-CoV-2 [3, 4]. ACE2 was originally identified as the receptor for SARS-CoV in the African green monkey kidney cell line VeroE6 [3]. During the COVID-19 pandemic, it was reported by several groups that SARS-CoV-2 also depends on ACE2 for entry into host cells [4–7]. While these studies have provided definitive evidence that ACE2 is a receptor for both SARS coronaviruses, whether it is the functional receptor in target respiratory epithelial cells remains to be verified.

Knowledge about cellular localization and abundance of SARS-CoV receptors is critical for validation of their roles as functional receptors and therapeutic targets in the pathogenesis of COVID-19. Accordingly, there has been a rapidly growing body of literature on ACE2 expression in normal and pathological lung tissues during the COVID-19 pandemic. In the majority of these studies, transcriptomic datasets were analyzed at a single-cell resolution [8–15] and showed only modest expression levels of ACE2 in the non-diseased airways. For example, in one study *ACE2* expression was detected in 6.7% of type II alveolar epithelial cells (AT2s) in healthy adult non-human primate (NHP) lungs and in 1.3% of secretory cells in human upper airway specimens [11]. In another study, RNA *in situ* mapping revealed the highest *ACE2* expression in the nose with decreasing expression throughout the lower respiratory tract [16]. However, at the protein level, studies have yielded conflicting results ranging from abundant expression of ACE2 in most epithelial cells to “no or only low level” expression in the airways [17–24].

The aim of this study was to determine the cellular localization and abundance of ACE2 protein in rodent and NHP models of lung injury and human respiratory tract with and without COVID-19 through a comparative and comprehensive analysis.

## Materials and methods

### Animal models

Frozen and formalin-fixed, paraffin-embedded (FFPE) NHP (*Papio papio*) lung tissue samples were provided by the Southwest Foundation for Biomedical Research (San Antonio, TX, USA) [25, 26]. All procedures performed on these animals were reviewed and approved by the Institutional Animal Care and Use Committees (IACUC) of the Southwest Foundation for Biomedical Research and the University of Texas Southwestern Medical Center. Briefly, baboons that were delivered by hysterotomy at 125 days (~ 27 weeks human gestation equivalent) or 140 days (~ 30 weeks human gestation equivalent) and euthanized immediately served as the gestational controls (GC). Animals delivered *via* natural delivery at ~185 days (full-term gestation) and sacrificed after 2–3 days served as full-term controls. A cohort of animals delivered at 125 days gestation were intubated, treated with exogenous surfactant (Survanta, 100 mg/kg) and maintained on mechanical ventilation in a humidified, pressure-limited, time-cycled infant ventilator and *pro re nata* (PRN) oxygen for 14 days to induce pathologic and biochemical findings that are characteristic of the “new” bronchopulmonary dysplasia (BPD) as seen in human infants [25, 27]. Adult NHP (Papio papio) lung samples comprised of necropsy tissue from healthy animals aged between 6 and 9 years.

Frozen and FFPE lung tissues from NHP (*Cynomolgus macaque*) model of cigarette-smoke exposure-induced COPD were from the Primate Facility at the Lovelace Respiratory Research Institute (Albuquerque, NM). All *C*. *macaque* used in the study were females, with average age of 11 years and a standard deviation of ±1 year. The NHPs were housed socially with up to 2 animals in a cage and exposed to 100% freshly filtered before initiating the exposures. For cigarette-smoke (CS) exposure, animals were kept in H2000 whole body chambers and provided 250 mg/m^3^ total suspended particulate matter of CS for 6 hours in a day/5 times a week to mimic human smoking ~1.8 to 4 cigarette packs in a day [28]. These experiments were performed with approval from the IACUC of Lovelace Respiratory Research Institute (Albuquerque, NM). F344/N rat tissues were also procured with approval from the IACUC of Lovelace Respiratory Research Institute (Albuquerque, NM) [29].

All murine studies were performed at Brigham and Women’s Hospital and approved either by Harvard Medical School or Brigham and Women’s Hospital IACUC. For hyperoxia-induced lung injury, adult (6–8 wk old) C57BL/6 mice were subjected to 21% (room air) or 95% oxygen for 72 h as previously described [30]. For the bacterial pneumonia model, C57BL/6 mice were given 50 μl intranasal *Pseudomonas aeruginosa* S470 strain (2 x 10^8^ CFU) or sterile PBS under isoflurane anesthesia and euthanized at 24 h post-infection. Right lung was inflated with 10% formalin and embedded in paraffin as previously described [31]. For ovalbumin-induced allergic airway model, SvEv129 X C57BL/6 WT mice were sensitized with a low dose (10 μg) of ovalbumin diluted in 200 μl of PBS along with 1 mg of alum by the i.p. route on days 0 and 7. Mice were then challenged with an aerosolized 6% solution of endotoxin-free ovalbumin in PBS on days 14–17. For all studies, control mice were sensitized with i.p. PBS and challenged with aerosolized PBS. Twenty four hours after the last ovalbumin or PBS challenge, mice were euthanized, lungs were inflated to 25 cm H2O pressure, fixed in formalin, embedded in paraffin as previously described [32].

### Human tissues

FFPE biopsy and autopsy specimens were retrieved from the Brigham and Women’s Hospital (BWH) Department of Pathology archives and obtained with the approval of the Institutional Review Board at BWH. The human samples used in this study were completely de-identified before researchers accessed the samples.

### Immunohistochemistry (IHC) and immunofluorescence (IF)

IHC was performed as previously described [33, 34]. For mouse, rat and NHP tissues, IHC was performed on a minimum of three cases per group unless indicated otherwise. Information on human tissues is provided in S2 Table. Each FFPE specimen was stained with H&E or Alcian Blue-H&E for histopathological assessment. 5μm thin sections were deparaffinized with xylene and then hydrated with graded ethanol. Antigen retrieval was performed using the Antigen Unmasking Solution (H3300, Vector Laboratories, Burlingame, CA) at 95˚C for 15 min. Sections were blocked by incubating with 10% normal serum from appropriate host diluted in PBS with 2% bovine serum albumin (Sigma Aldrich; St. Louis, MO) for 60 min. Primary antibody incubations were performed overnight at 4˚C at the dilutions indicated in S1 Table. Omission of the primary antibody, use of an irrelevant antiserum (anti-GFP antibody), and rabbit IgG isotype antibodies served as negative controls. Positive controls for NHP and human lung tissues included anti-pro-surfactant protein C (SPC) and anti- α -tubulin antibodies. After the primary antibody incubation, sections were incubated with a biotinylated secondary antibody, which was detected by incubation for 30 min with an avidin–biotin–peroxidase complex (Vectastain ABC kit; Vector Laboratories), followed by a 10-min PBS wash, then incubation with a 3,3-diaminobenzidine substrate/chromogen solution (DAB Peroxidase Substrate Kit; Vector Laboratories). Sections were counterstained with hematoxylin, viewed under a Nikon Eclipse 80*i* microscope, and images were captured using NIS-Elements Basic Research® software. Immunostaining results were scored in a blinded fashion, whenever possible, by two independent observers (SC and JLH). Key results were confirmed by IHC performed in the Immunohistochemistry Laboratory in the Department of Pathology at BWH using the Novolink Polymer Detection System (Leica Biosystems, Buffalo Grove, IL) [35]. Double IF analysis was performed as previously described [36]. Briefly, following primary antibody incubations overnight at 4˚C, sections were incubated with fluorescence-conjugated secondary antibodies (Alexa Fluor® 594 goat anti-mouse IgG and Alexa Fluor® 488 goat anti-rabbit IgG, Thermo Fisher Scientific). After washing in PBS, sections were mounted in Vectashield (Vector Laboratories) for nuclei counterstaining.

### Immunoblotting

Immunoblotting analysis was performed as previously described [37]. Briefly, tissues were homogenized on ice in RIPA buffer (Thermo Fisher Scientific) containing protease inhibitor cocktail (Roche, Basel, Switzerland). Homogenate was centrifuged at 4°C for 15 minutes at 12,000 rpm and the supernatant was used for protein concentration determination using BCA kit (Thermo Fisher Scientific). Equal amounts of total protein (30 μg) were reduced by adding Laemmli sample buffer containing dithiothreitol and heating to 90˚C for 5 minutes. Proteins in the samples were separated on 12.5% sodium dodecyl sulfate (SDS)- polyacrylamide gels, then transferred to nitrocellulose membranes, blocked in phosphate-buffered saline (PBS) containing 5% nonfat milk and 0.1% Tween-20 (PBS/T) and incubated overnight with ACE2 or TMPRSS2 antibodies at the dilutions indicated in S1 Table. After incubations with the secondary antibodies and chemiluminescence substrate (SuperSignal^TM^ West Pico PLUS, Thermo Scientific, Waltham, MA), protein bands were detected by autoradiography. Signals were quantified using densitometry with Image-J software (NIH, Bethesda, MD) and normalized to β-actin (ACTB) signals.

### Statistical analysis

Densitometry results were analyzed using one-way ANOVA followed by Tukey’s multiple comparisons test (Prism 8, GraphPad Software Inc., San Diego, CA).

## Results

### ACE2 expression is not detected in NHP lungs with and without cigarette smoke-induced airway injury

**Fig 1:**
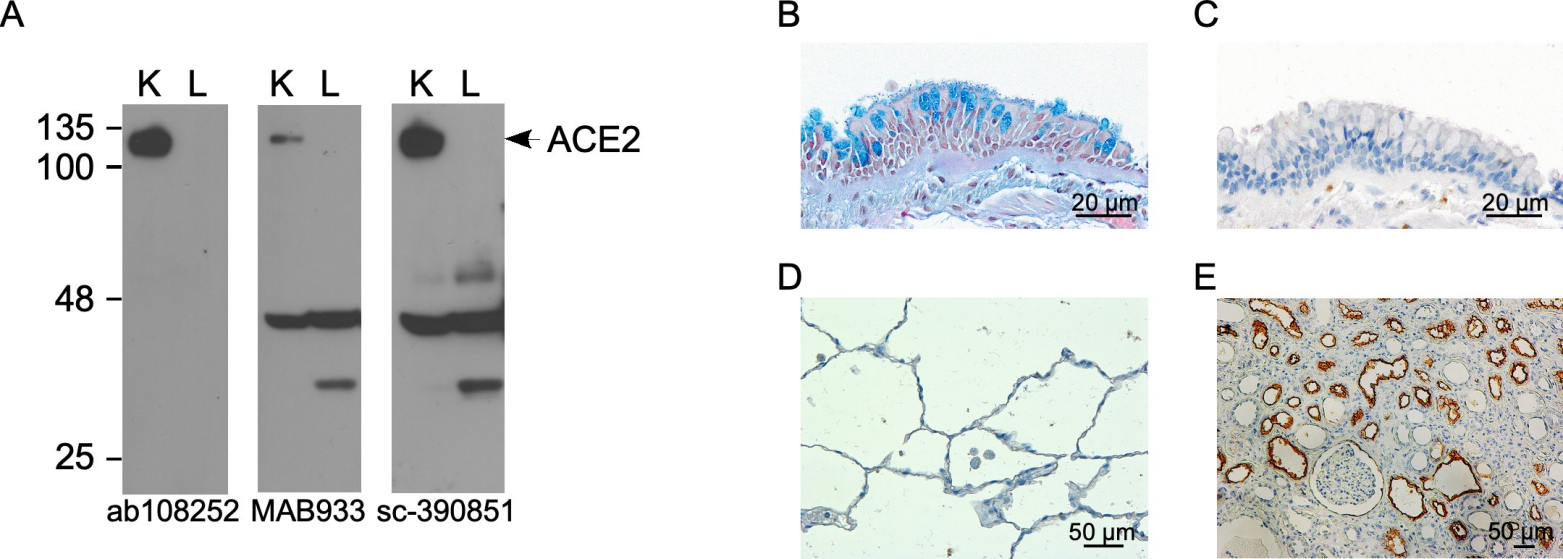
ACE2 expression is not detected at baseline and not induced by cigarette-smoke exposure or bronchopulmonary dysplasia in adult NHP lungs. Representative immunoblots with kidney and lung homogenates (30 mg/lane) from *C*. *macaque* model of cigarette-smoke-induced airway injury; identical membranes were probed with 3 different ACE2 antibodies as indicated on the image. K, kidney; L, lung (A). Representative image of lung section from *C*. *macaque* model of cigarette-smoke-induced airway injury stained with Alcian Blue (AB) and H&E demonstrating abundant goblet cells in the bronchial epithelium (B), but lack of ACE2 immunoreactivity in bronchial (C) and alveolar epithelial cells (D). A human kidney section was used as a positive control and demonstrates abundant ACE2 expression in proximal tubular epithelial cells (E).

Cigarette smoke exposure has been shown to increase *ACE2* mRNA expression levels in the human lung [8, 9]. We first assessed ACE2 expression in a *C*. *macaque* model of cigarette smoke-induced airway injury by immunoblotting [28]. Three different commercially available ACE2 antibodies were tested (S1 Table) and all demonstrated an ACE2 band around 105 kDa in the positive control kidney homogenate, but not in lung homogenates (Fig 1A). Two of the antibodies (MAB933 and sc-390851) also demonstrated lower molecular weight (MW) bands in addition to the 105 kDa band in all samples, while one antibody (ab108252) did not. We considered the possibility that these bands might represent the recently described novel and truncated ACE2 isoform, which is induced by interferons and viruses [38]. However, IHC of *C*. *macaque* lung sections using ab108252, which did not detect the lower MW bands, and sc-390851, which detected the lower MW bands, revealed similar results with absence of ACE2-expressing cells in both control and cigarette smoke-exposed groups (Fig 1B–1D), thus suggesting that these bands were most likely non-specific. In contrast, IHC with both antibodies demonstrated robust expression of ACE2 in the kidney in the apical surface of the proximal tubular epithelium (Fig 1E). Thus, ACE2 protein was undetectable in control and cigarette-smoke exposed *C*. *macaque* lungs by immunoblotting and IHC.

### Expression of ACE2 is higher and TMPRSS2 is lower in preterm NHP lungs compared to full-term and adult NHP lungs

**Fig 2:**
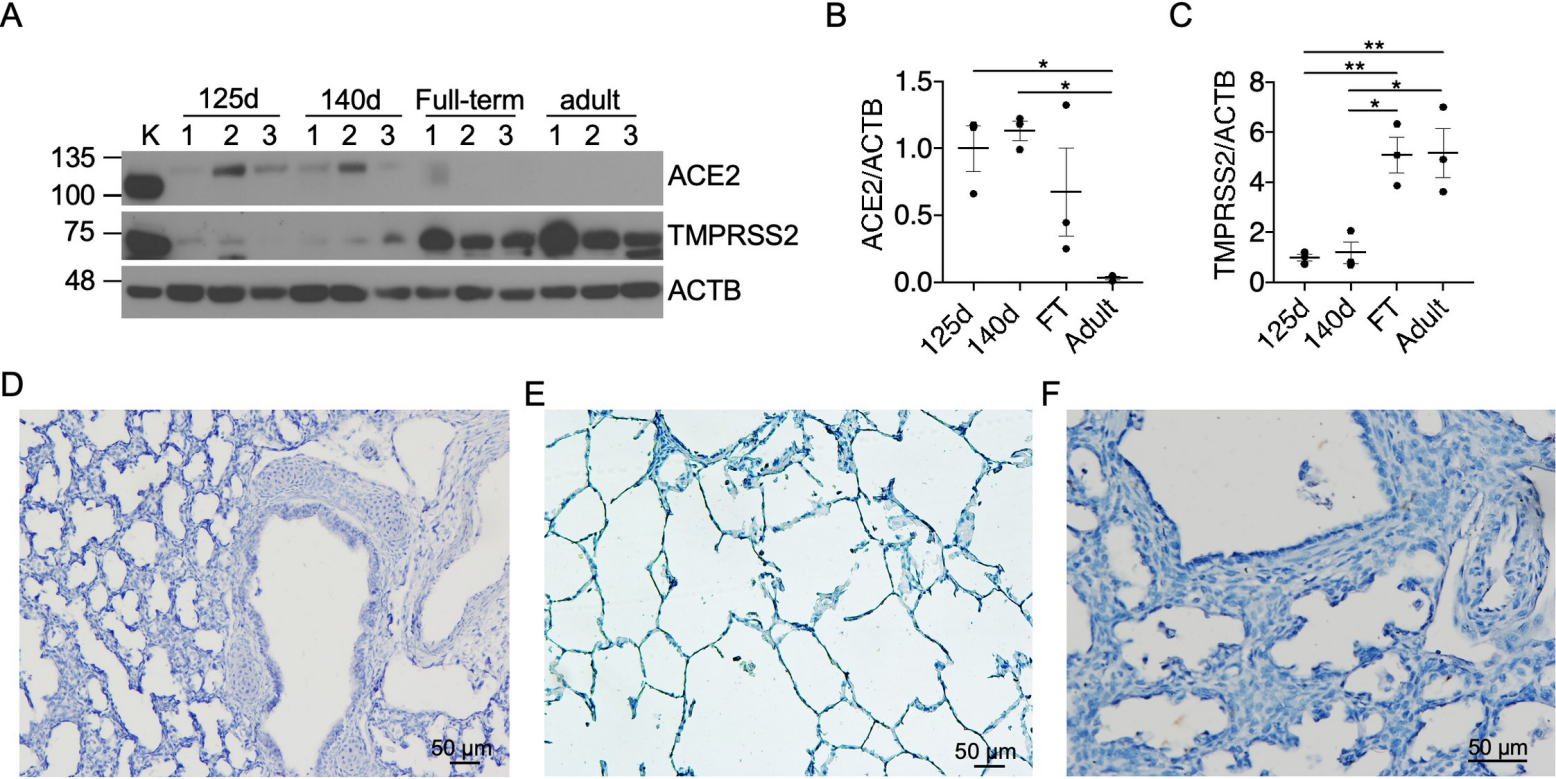
Expression of ACE2 is higher and TMPRSS2 is lower in preterm lungs compared to full-term and adult lungs. Representative immunoblots and densitometry of NHP (*P*. *papio*) lung homogenates from preterm (125d and 140d) and full-term newborn and adult animals demonstrate faint ACE2 expression in the 125d and 140d groups, but not in adult samples (A & B), whereas TMPRSS2 expression is significantly higher in adult lungs compared to other groups (A & C). Representative images of IHC for ACE2 in NHP (*P*. *papio*) lung tissues from preterm (125d) (D) and adult animals (E) as well as NHP lungs with BPD (F) demonstrate lack of ACE2 immunoreactivity. * *p* < 0.05, ** *p* < 0.01.

*In utero* vertical transmission of COVID-19 during pregnancy and severe COVID-19 in newborns have been documented only in rare cases [39]. To determine whether the relative protection of fetus and newborns could be due to a developmental delay in ACE2 expression, we examined ACE2 expression in NHP (*P*. *papio*) lungs first by immunoblotting. There were faint ACE2 bands in lung homogenates from preterm animals delivered at 125 days (~ 27-wk human gestation) and 140 days (~ 30-wk human gestation) of gestation, but not from full-term newborn or adult NHPs (Fig 2A and 2B). In contrast to ACE2, TMPRSS2 expression demonstrated a significant increase in full-term and adult lung samples in comparison to preterm lung samples (Fig 2A and 2C). Lack of ACE2 expression in preterm newborn (Fig 2D) and adult lungs (Fig 2E) was confirmed by IHC. Furthermore, ACE2 immunoreactivity was not detected in the NHP model of BPD induced by mechanical ventilation and oxygen exposure following preterm delivery (Fig 2F).

### ACE2 protein is abundant in the upper and scarce in the lower airways in human lungs

**Fig 3:**
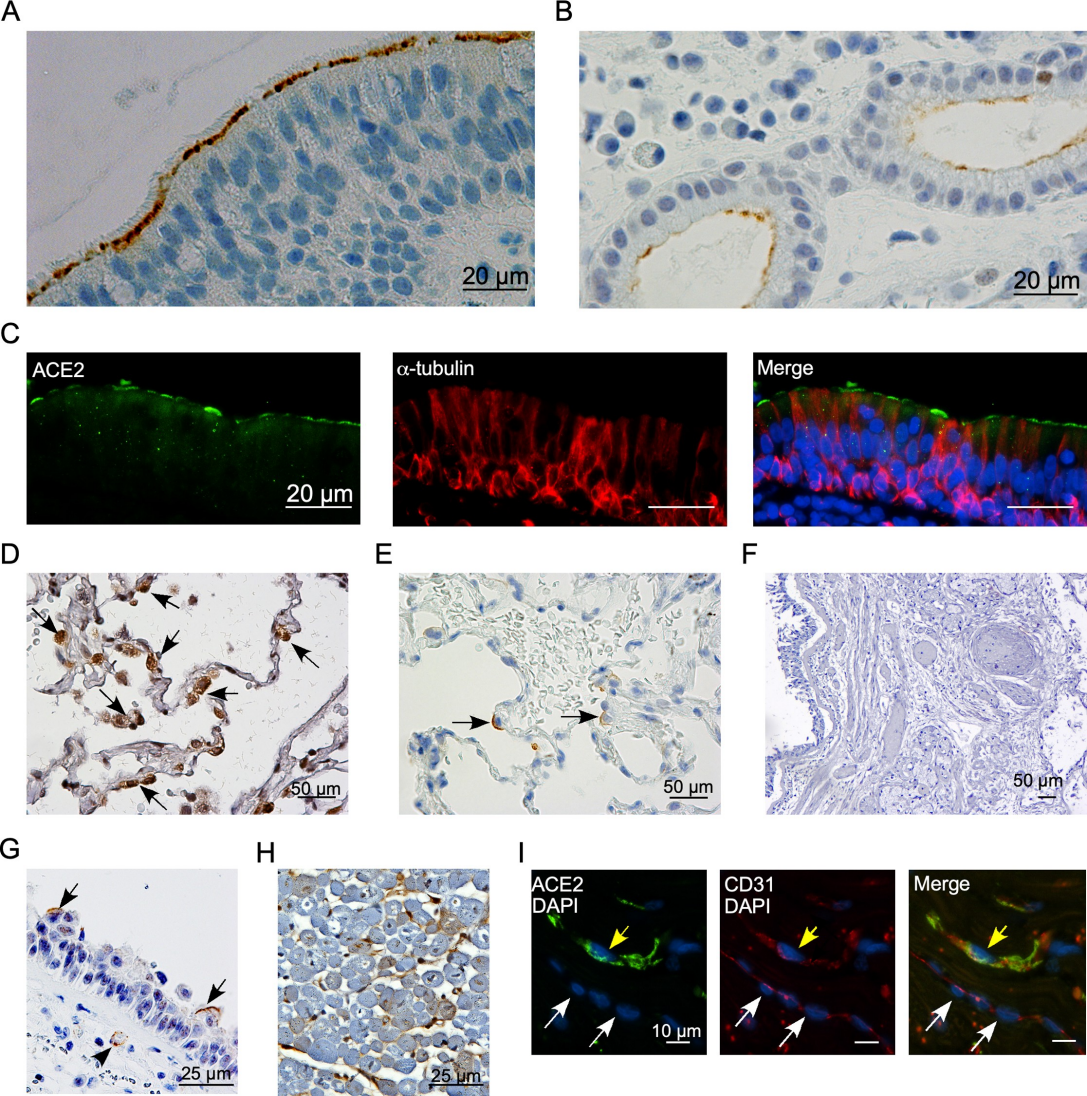
Robust ACE2 detection in ciliated epithelial cells in the upper but not lower airways in human specimens. Representative images of ACE2 IHC on control human paranasal sinus section showing ACE2 immunoreactivity in ciliated epithelial cells (A) and submucosal glandular epithelial cells (B). Double IF analysis confirmed co-localization of ACE2 in the ciliated epithelial cells with α-tubulin (C). Representative IHC for pro-SPC (D) and ACE2 (E) on control human lung sections; black arrows indicate pro-SPC positive AT2s in panel “D” and ACE2-positive AT2s in panel “E”. Representative IHC for ACE2 on an autopsy lung section (F) and trachea (G) from patients who died of COVID-19. Black arrows indicate ACE2-positive ciliated epithelial cells and black arrowhead indicates a capillary blood vessel with ACE2 immunoreactivity in the lamina propria. Representative IHC for ACE2 (H) and double IF for ACE2 and CD31 (I) on control heart sections; yellow arrow indicates a capillary blood vessel with ACE2 and CD31 co-localization and white arrows indicate a larger blood vessel without ACE2 signal.

Next we examined ACE2 expression in a cohort of human biopsy and autopsy specimens by IHC and double IF analysis (S2 Table). In the upper respiratory tract, paranasal sinus mucosa biopsy specimens from 20 individuals demonstrated variable ACE2 immunoreactivity, ranging from 1+ to 4+, in a heterogenous pattern on the apical membrane of ciliated epithelial cells (Fig 3A) and epithelial cells of some submucosal glands (Fig 3B). Double IF analysis using antibodies against ACE2 and α-tubulin confirmed localization of ACE2 to ciliated epithelial cells (Fig 3C). In contrast, in histologically normal lung specimens with a normal abundance of AT2s as demonstrated with pro-surfactant protein C immunoreactivity (Fig 3D), ACE2 expression was almost completely absent in all cell types except for rare AT2s in 2 of the 10 specimens tested (Fig 3E).

### ACE2 is detected in the trachea and myocardium but not lung cells in fatal COVID-19 pneumonia

In autopsy specimens from 5 patients with COVID-19, ACE2 expression was not observed in any cells in the lung (Fig 3F), but was present in rare epithelial and microvascular endothelial cells in the trachea (Fig 3G). ACE2 expression was also examined in the heart, another major target organ of SARS-CoV-2, where ACE2 immunoreactivity was detected in association with myocardial microvasculature in both control (Fig 3H) and COVID-19 specimens (S1 Fig). Double IF analysis using antibodies against ACE2 and CD31, a marker of vascular endothelial cells, confirmed co-localization of ACE2 with CD31 in some microvascular endothelial cells (Fig 3I).

### ACE2 expression is induced in murine lungs with hyperoxia exposure and bacterial pneumonia

**Fig 4:**
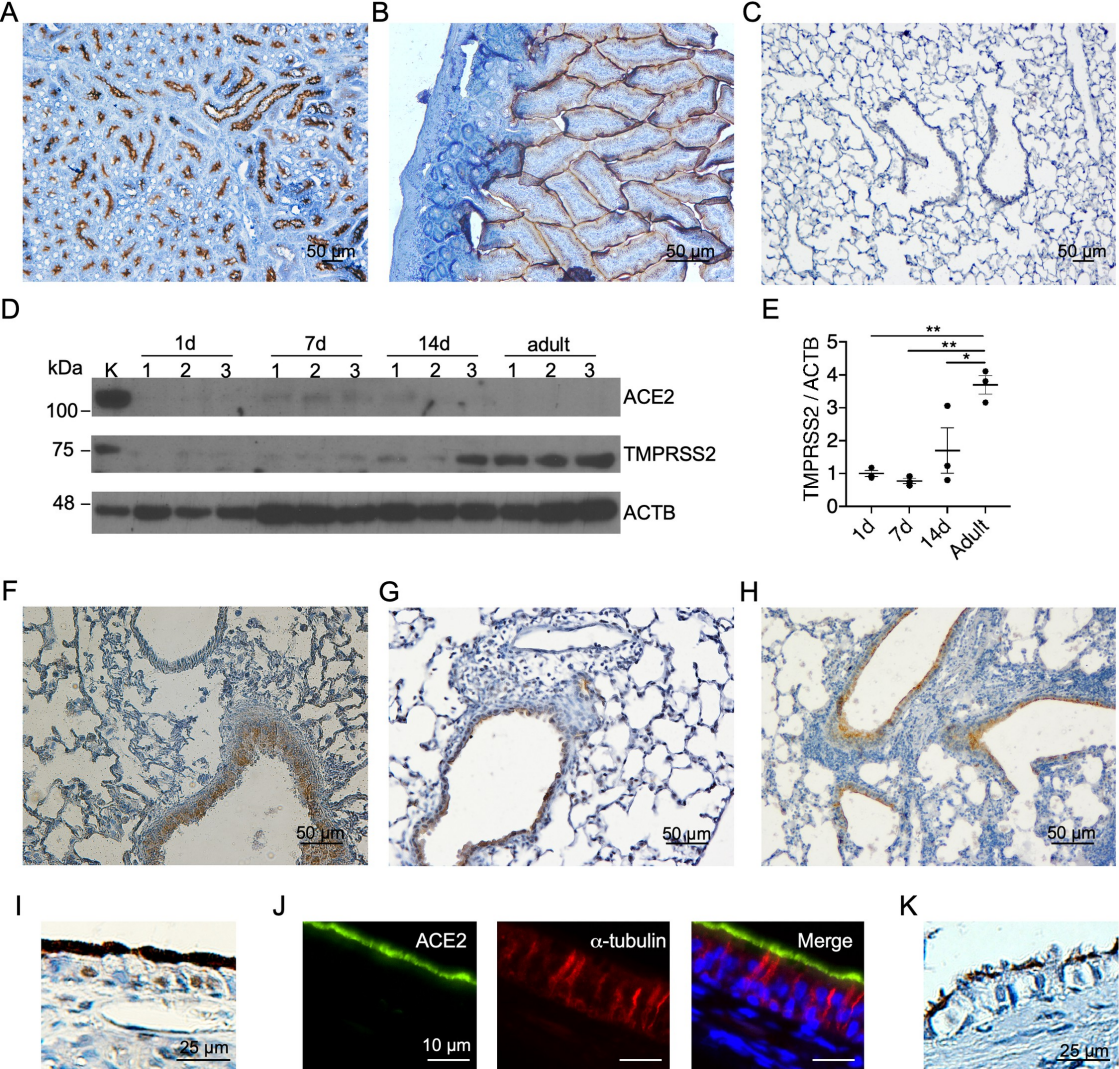
ACE2 is constitutively expressed in F433/N rat nasal mucosa and lungs, and induced in murine lungs with injury. Representative IHC for ACE2 in adult murine kidney (A), intestine (B), and lung (C). Representative IHC for ACE2 (J) and double IF for ACE2 and α-tubulin on F433/N rat nasal mucosa sections (K) and IHC for ACE2 on F433/N lung section (L). Immunoblotting and densitometry of murine whole lung homogenates from newborn and adult mice demonstrate TMPRSS2, but not ACE2 expression in adult samples (D & E). Representative adult murine lung sections with acute lung injury induced by hyperoxia exposure (F) and *P*. *aeruginosa* pneumonia (G), and ovalbumin-induced allargic airway inflammation (H) demonstrate diffuse ACE2 immunoreactivity in bronchial epithelial cells. Representative IHC for ACE2 (I) and double IF for ACE2 and α-tubulin on F433/N rat nasal mucosa sections (J) and IHC for ACE2 on F433/N lung section (K).

IHC with two different primary antibodies (ab108252 and PA5-47488) demonstrated similar results with robust ACE2 immunoreactivity along the apical and lateral border of proximal tubules in the murine kidney (Fig 4A). As expected, murine intestinal epithelial cells also demonstrated strong ACE2 immunoreactivity along the luminal border (Fig 4B). In contrast, neither antibody detected ACE2 immunoreactivity in newborn (not shown) or adult (Fig 4C) murine lung tissues. To confirm lack of ACE2 expression in murine lungs, we performed immunoblot analysis which demonstrated ACE2 expression in the control kidney homogenate, but not in whole lung homogenates from 1-d-old, 7-d-old, 14-d-old, or adult mice (Fig 4D). In contrast, consistent with the data from NHP lungs and a recent paper that was published during the preparation of this manuscript [40], TMPRSS2 expression was detected in lungs from all age groups in a pattern consistent with developmental upregulation, with detection of the highest TMPRSS2 levels in adult lungs compared to newborn lungs (Fig 4D and 4E).

Supplemental oxygen is administered to more than 75% of hospitalized patients with COVID-19 [41]. To determine whether hyperoxia exposure induced ACE2 expression in the lung, we performed IHC on lung sections of mice with hyperoxia-induced lung injury [37, 42]. In contrast to control lung samples, diffuse cytosolic ACE2 immunoreactivity was detected in bronchial epithelial cells in adult murine lungs exposed to 95% oxygen for 72 h (Fig 4F). Similarly, in lung sections from murine model of acute lung injury induced by *Pseudomonas aeruginosa* pneumonia (Fig 4G) [31] and ovalbumin-induced allergic airway inflammation (Fig 3H) [32], ACE2 expression was detected in bronchial, but not alveolar epithelial cells by IHC [31].

### ACE2 expression in F344/N rat nasal mucosa and lungs recapitulate ACE2 expression in the human sinonasal epithelium

IHC and double IF for α-tubulin and ACE2 demonstrated robust and uniform expression of ACE2 in ciliated epithelial cells in F344/N nasal mucosa specimens (Fig 4I and 4J). Furthermore, in contrast to murine, NHP, and human lungs, rat lungs displayed immunoreactivity for ACE2 in the ciliated airway epithelial cells (Fig 4K).

## Discussion

A thorough understanding of the pathogenesis of COVID-19 requires knowledge of *in vivo* sites of interaction between the virus and host cells. In this study, we aimed to fill the current knowledge gap on constitutive and induced expression patterns of ACE2 protein in the respiratory tract by leveraging tissue samples from rodent and NHP models as well as humans with and without COVID-19. Despite a plethora of studies showing ACE2 mRNA expression in various cell types in the human lung, there is controversy on the distribution and abundance of ACE2 protein expression, which has critical importance in understanding how SARS-CoV-2 infection spreads to the lung [18, 19, 21, 23]. In this study, we first performed a detailed characterization and optimization of a panel of ACE2 antibodies that were used in previously published studies. Our antibody panel included antibodies directed against the extracellular domain (ab108252), carboxy terminus (sc-390851), as well as a longer peptide including both amino and carboxy terminal residues (MAB933). All three antibodies were first titrated using human kidney tissue, which is known to express high levels of ACE2. The “secondary-only” controls and IF were used to identify chromogen-related artifacts. Subsequently, ACE2 expression was assessed on each tissue type using a minimum of two of the ACE2 antibodies. Following this rigorous approach, we detected strong ACE2 expression in the ciliated epithelium of the paranasal sinuses, a continuum of the nasal epithelium, in 95% of the cases examined. However, when we applied the same reagents and methods used in IHC of sinus specimens to lung tissues, we were unable to detect ACE2 expression in the latter group with the exception of rare AT2s (5–10 cells per slide) in only 20% of the cases examined. Thus, although our results demonstrate more common and robust ACE2 staining in the sinonasal ciliated epithelium than those reported by Hikmet *et al*, they are in agreement with their finding on lack and scarcity of ACE2 immunoreactivity in the bronchial epithelium and AT2s, respectively [18]. In contrast, we were not able to confirm the abundant expression of ACE2 in ciliated bronchial epithelial cells or AT2s as recently reported by Lee *et al*. and Wijnant *et al*., respectively [19, 24]. Notably, in both of these studies, a polyclonal rabbit antisera (ab15348, Abcam), which was raised against a synthetic peptide corresponding to 20 amino acids at the C terminal of human ACE2 was employed. While our finding of a significant gradient in ACE2 protein expression between upper and lower airways differs from these two studies, it is in complete agreement with the gradient of *ACE2* mRNA and SARS-CoV-2 infection recently described by Hou *et al*. [16]. Notably, we also identified high expression levels of ACE2 in F344/N rat nasal mucosa and lungs, which may constitute a convenient *in vivo* model for SARS-CoV-2, provided rat ACE2, unlike murine ACE2, can engage the virus [5, 43].

One possibility that could underlie the lack of *in situ* ACE2 detection in control human lung tissues is shedding of ACE2 in the protease-rich lung milieu. To investigate this possibility, we analyzed lung homogenates from two different NHP models by immunoblotting. These studies demonstrated weak expression of ACE2 only in preterm, but not full-term or adult *P*. *papio* lungs. However, we did not detect any ACE2 immunoreactivity by IHC on FFPE preterm *P*. *papio* lungs. Thus, it is conceivable that the higher sensitivity of immunoblotting compared to IHC may be reflective of some ACE2 shedding in the airways in preterm lungs and needs to be investigated further. On the other hand, immunoblotting and IHC results both showed lack of ACE2 immunoreactivity in the *C*. *macaque* model of cigarette smoke-induced airway injury. This result differs from significant ACE2 upregulation reported by Liu *et al*. in human lung specimens of cigarette smokers and may be explained by the relative lack of chronicity in the NHP model [44]. However, consistent with our findings, images of human airways from non-smokers in the paper by Liu *et al*. demonstrate lack of ACE2 immunoreactivity.

We did not detect any ACE2 immunoreactivity in autopsy lung specimens from a small cohort of patients with COVID-19. Although initial studies have suggested that *ACE2* is an interferon-inducible gene [11], a recent study has shown this to be the case only for a truncated isoform of *ACE2* that does not bind SARS-CoV-2 [38]. Thus, it is highly unlikely that the SARS-CoV-2-binding isoform of ACE2 would be up-regulated by type I interferons during COVID-19. In mice, we found that hyperoxia exposure induced ACE2 expression in bronchial epithelial cells. It is thus plausible that supplemental oxygen treatment during early stages of COVID-19 could up-regulate ACE2 expression in the lower airways, which may not be captured in autopsy specimens due to diffuse lung damage with loss of AT2s and bronchial epithelial cells. Furthermore, examination of COVID-19 specimens from larger cohorts are needed to determine the extent to which COVID-19 affects ACE2 expression in smoker lungs.

In conclusion, our findings demonstrate that while high levels of ACE2 protein are localized to the ciliated epithelium in the upper airways, constitutive ACE2 expression is scarce and in most cases below the detection level of IHC and immunoblotting in adult NHP and human lungs. We did not find evidence of induced ACE2 expression in NHP lungs with injury or human lungs with COVID-19. While these data unequivocally support the key role of ACE2 in the sinonasal mucosa as a functional receptor of SARS-CoV-2, the restricted baseline expression pattern of ACE2 in the lung presents a conundrum as it raises questions about alternative viral receptors or additional mechanisms of lung injury in COVID-19 pneumonia.

## Supporting information

S1 Fig ACE2 expression in an autopsy heart specimen from a patient with COVID-19. Representative IHC for ACE2 on an autopsy heart specimen from a patient with COVID-19 demonstrates ACE2 immunoreactivity associated with microvasculature in the myocardium (black arrows).
(TIF)

S1 Table Description of primary antibodies used for IHC/IF and western blotting (WB). (PDF)

S2 Table Characteristics for the human specimens used and semi-quantitative assessment of ACE2 abundance based on immunohistochemistry. Semi-quantitative assessment of ACE2 abundance was based on the following scale: 1+, individual cells; 2+, one focus with a cluster of cells (> 10 cells); 3+, multiple foci of clusters of cells; 4+, multiple foci of clusters of cells with intense staining. EC, epithelial cells; mEC, microvascular endothelial cells.
(PDF)

S1 Raw images (PDF)
