# Supplementary figures and images for: Comparative analysis of ACE2 protein expression in rodent, non-human primate, and human respiratory tract at baseline and after injury: A conundrum for COVID-19 pathogenesis
Source: PLoS One. 2021 Feb 24;16(2):e0247510. doi: 10.1371/journal.pone.0247510 (PMC7904186; doi:10.1371/journal.pone.0247510)

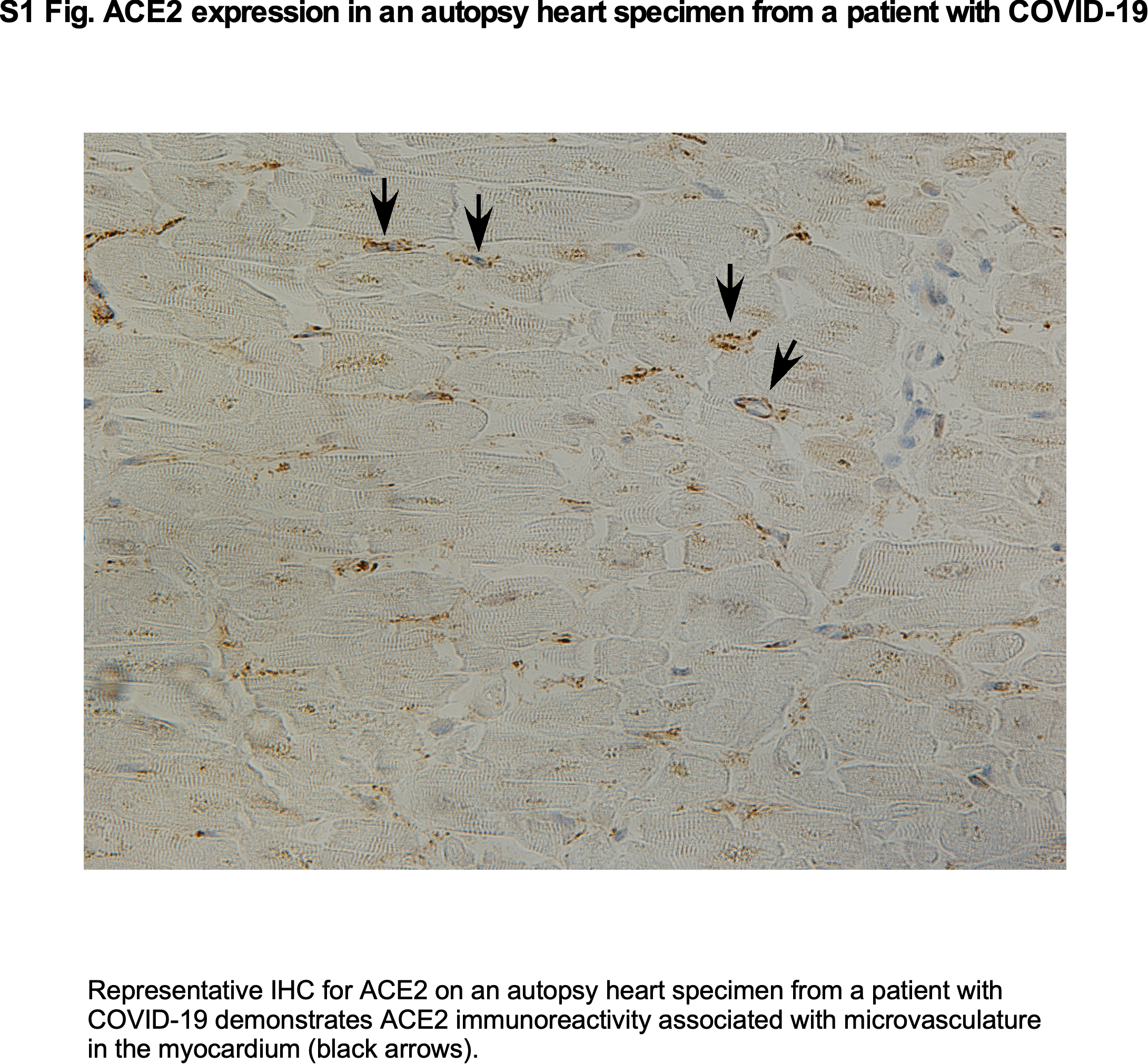

Supplement: S1 Fig — Representative IHC for ACE2 on an autopsy heart specimen from a patient with COVID-19 demonstrates ACE2 immunoreactivity associated with microvasculature in the myocardium (black arrows). (TIF) [file pone.0247510.s001.tif]

Full unedited gel for Fig 1A

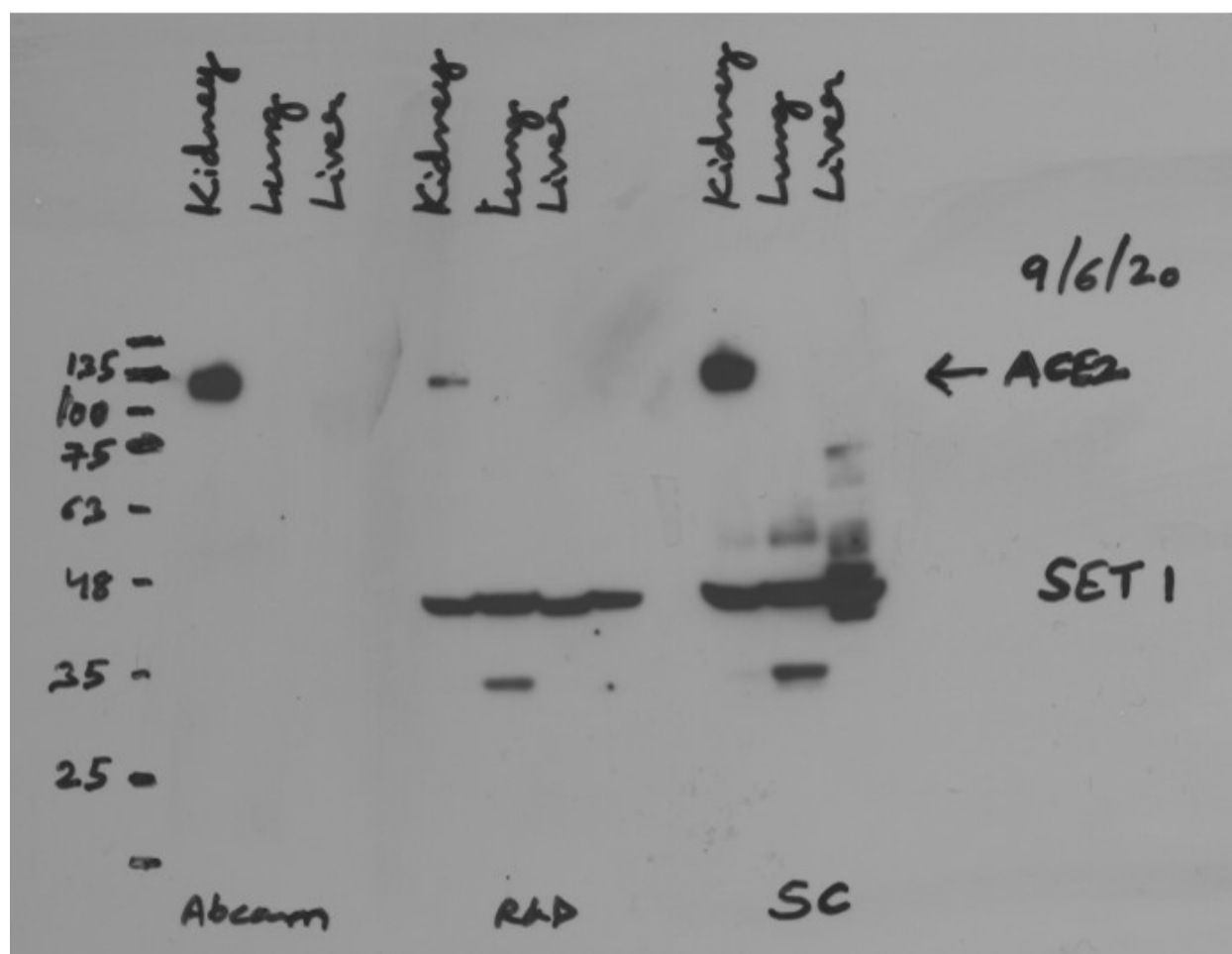

Full unedited gel for Fig 2A

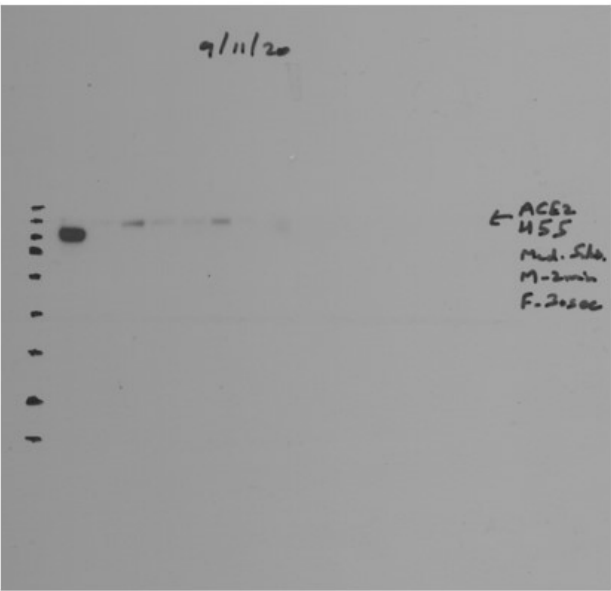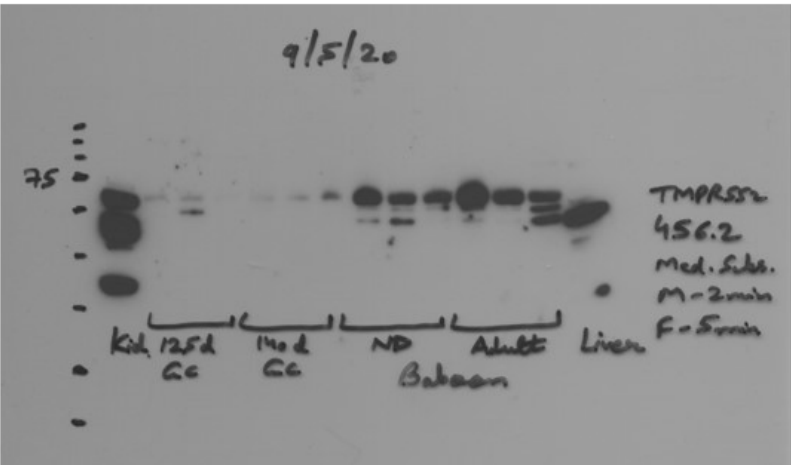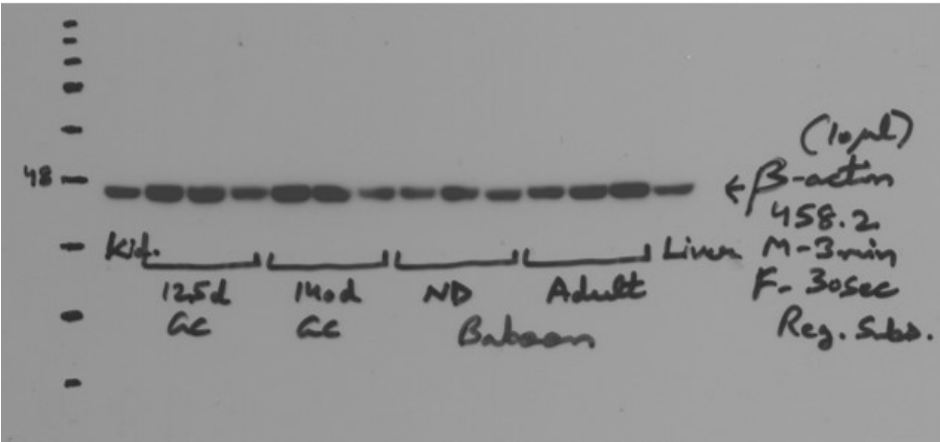

Full unedited gel for Fig 4D

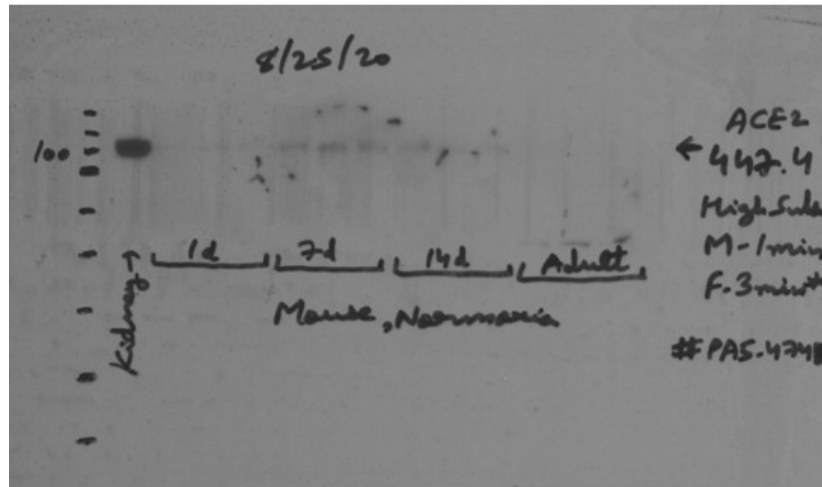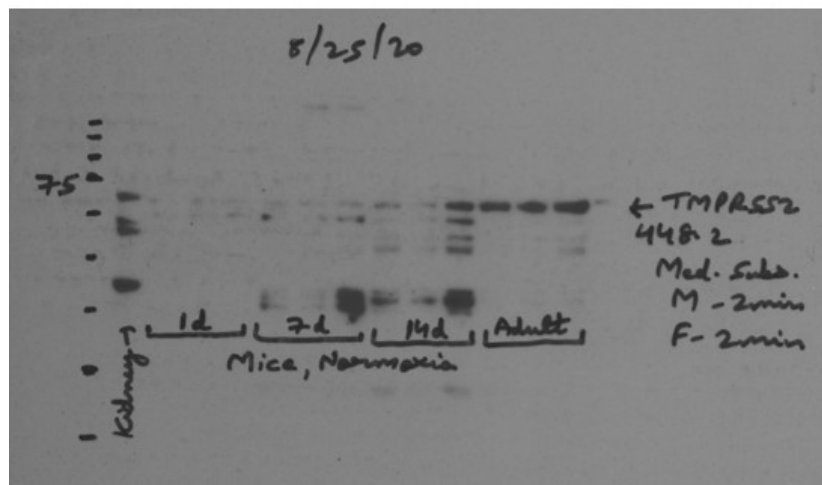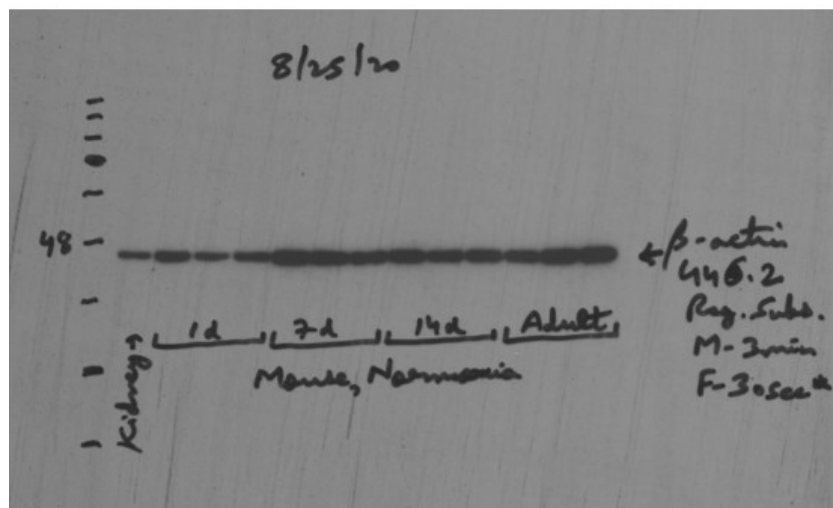

Supplement: S1 Raw images — (PDF) [file pone.0247510.s004.pdf]
