# Supplementary material for: Comparative analysis of ACE2 protein expression in rodent, non-human primate, and human respiratory tract at baseline and after injury: A conundrum for COVID-19 pathogenesis
Source: PLoS One. 2021 Feb 24;16(2):e0247510. doi: 10.1371/journal.pone.0247510 (PMC7904186; doi:10.1371/journal.pone.0247510)
Supplement: S1 Table — (PDF) [file pone.0247510.s002.pdf]

S1 Table. Description of primary antibodies used for IHC/IF and western blotting (WB)

Antibody (Clone)	Manufacturer	Catalogue No.	Host	Class	Immunogen	Application	Dilution
ACE2 (E-11)	Santa Cruz Biotechnology	sc-390851	Mouse	Monoclonal	Amino acids 631-805 of ACE2 of human origin	WB	1:100
ACE2 (EPR4435(2))	Abcam	ab108252	Rabbit	Monoclonal	Synthetic peptide within human ACE2 aa 200-300 (extracellular)	IHC/IF<br>WB	1:150<br>1:1000
ACE2 (171606)	R&D Systems	MAB933	Mouse	Monoclonal	Mouse myeloma cell line NS0-derived recombinant human ACE-2 Gln18-Ser740	IHC/IF<br>WB	1:100<br>1:250
ACTB (β-Actin) (AC-15)	Invitrogen,	AM4302	Mouse	Monoclonal		WB	1:5000
CD31 (JC70A)	Dako	M0823	Mouse	Monoclonal		IF	1:40
GFP	Invitrogen	A-11122	Rabbit	Polyclonal		IHC	1:100
SPC (Prosurfactant Protein C)	Abcam	ab90716	Rabbit	Polyclonal		IF	1:100
TMPRSS2 (H-4)	Santa Cruz Biotechnology	sc-515727	Mouse	Monoclonal		WB	1:100
α-Tubulin (B-7)	Santa Cruz Biotechnology	sc-5286	Mouse	Monoclonal		IF	1:300
