# Supplementary material for: Comparative analysis of ACE2 protein expression in rodent, non-human primate, and human respiratory tract at baseline and after injury: A conundrum for COVID-19 pathogenesis
Source: PLoS One. 2021 Feb 24;16(2):e0247510. doi: 10.1371/journal.pone.0247510 (PMC7904186; doi:10.1371/journal.pone.0247510)
Supplement: S2 Table — Semi-quantitative assessment of ACE2 abundance was based on the following scale: 1+, individual cells; 2+, one focus with a cluster of cells (> 10 cells); 3+, multiple foci of clusters of cells; 4+, multiple foci of clusters of cells with intense staining. EC, epithelial cells; mEC, microvascular endothelial cells. (PDF) [file pone.0247510.s003.pdf]

**S2 Table. Characteristics for the human specimens used and semi-quantitative assessment of ACE2 abundance based on immunohistochemistry***

| Anatomic location | Age | Gender | Diagnosis/description | ACE2 abundance, location |
|---|---|---|---|---|
| Paranasal sinus | 27 | F | Sinusitis (minimal inflammation) | none |
| | 59 | M | Sinusitis (marked inflammation) | 1+, ciliated EC |
| | 51 | M | Sinusitis (marked inflammation) | 4+, ciliated EC |
| | 65 | M | Sinusitis (marked inflammation) | 3+, ciliated EC |
| | 57 | F | Sinusitis (minimal inflammation) | 1+, ciliated EC |
| | 45 | F | Sinusitis (minimal inflammation) | 2+, ciliated EC |
| | 24 | M | Sinusitis (minimal inflammation) | 1+, ciliated EC |
| | 45 | F | Sinusitis (marked inflammation) | 2+, ciliated EC |
| | 89 | M | Sinusitis (low inflammation) | 4+, ciliated EC; 1+, submucosal glands |
| | 72 | F | Sinusitis (marked inflammation) | 4+, ciliated EC; 1+, submucosal glands |
| | 25 | M | Sinusitis (minimal inflammation) | 1+, ciliated EC; 1+ submucosal glands |
| | 52 | F | Sinusitis (marked inflammation) | 3+, ciliated EC; 2+ submucosal glands |
| | 52 | M | Sinusitis (marked inflammation) | 3+, ciliated EC |
| | 18 | F | Sinusitis (minimal inflammation) | 2+, ciliated EC |
| | 53 | F | Sinusitis (marked inflammation) | 1+, ciliated EC; 1+ endothelial cells |
| | 70 | M | Sinusitis (marked inflammation) | 4+, ciliated EC |
| | 58 | F | Sinusitis (marked inflammation) | 3+, ciliated EC |
| | 64 | F | Sinusitis (moderate inflammation) | 3+, ciliated EC; 1+, submucosal glands |
| | 27 | M | Sinusitis (moderate inflammation) | 2+, ciliated EC |
| | 30 | F | Sinusitis (moderate inflammation) | 3+, ciliated EC |
| Nasal cavity | 77 | F | Non-pathologic | 1+, ciliated EC |
| Nasopharynx | 66 | M | Non-pathologic | none |
| | 47 | M | Non-pathologic | none |
| | 69 | F | Non-pathologic | none |
| Trachea | 77 | M | COVID-19 | 1+, ciliated EC; 1+, mECs |

| | | | | |
|---|---|---|---|---|
| | 68 | F | COVID-19 | 2+, ciliated EC; 1+, mECs |
| | 66 | F | COVID-19 | 1+, ciliated EC; 1+, mECs |
| | 57 | M | COVID-19 | 1+, ciliated EC; 1+, mECs |
| | 50 | M | COVID-19 | 2+, ciliated EC; 1+, mECs |
| | 66 | M | COVID-19 | 1+, ciliated EC; 1+, mECs |
| | 53 | M | COVID-19 | 1+, ciliated EC; 1+, mECs |
| Lung | 58 | F | Non-pathologic | none |
| | 62 | M | Non-pathologic | none |
| | 63 | F | Non-pathologic | none |
| | 55 | M | Non-pathologic | none |
| | 72 | M | Non-pathologic | 1+, AT2s |
| | 48 | F | Non-pathologic | none |
| | 59 | F | Non-pathologic | none |
| | 63 | M | Non-pathologic | 1+, AT2s |
| | 65 | M | Non-pathologic | none |
| | 65 | M | Non-pathologic | none |
| | 68 | F | COVID-19 | none |
| | 50 | M | COVID-19 | none |
| | 66 | M | COVID-19 | none |
| | 53 | M | COVID-19 | none |
| | 57 | M | COVID-19 | none |
| Liver | 58 | F | Non-pathologic | 2+, biliary ductal EC; 1+ endothelial cells |
| | 62 | F | Non-pathologic | 2+, biliary ductal EC; 2+ endothelial cells |
| | 62 | M | Non-pathologic | 3+, biliary ductal EC; 1+ endothelial cells |
| Kidney | 70 | M | Non-pathologic | 4+, proximal tubular EC; 4+, parietal layer of glomerulus |
| | 70 | M | Non-pathologic | 4+, proximal tubular EC; 4+, parietal layer of glomerulus |
| | 86 | F | Non-pathologic | 4+, proximal tubular EC; 4+, parietal layer of glomerulus |

| | | | | |
|---|---|---|---|---|
| Heart | 70 | M | Non-pathologic (post-transplant) | 3+, mECs |
| | 53 | F | Non-pathologic (post-transplant) | 3+, mECs |
| | 68 | M | Non-pathologic (post-transplant) | 3+, mECs |
| | 68 | F | COVID-19 | 3+, mECs |
| | 66 | F | COVID-19 | 3+, mECs |
| | 57 | M | COVID-19 | 3+, mECs |

[*] Semi-quantitative assessment of ACE2 abundance was based on the following scale: 1+, individual cells; 2+, one focus with a cluster of cells (> 10 cells); 3+, multiple foci of clusters of cells; 4+, multiple foci of clusters of cells with intense staining. EC, epithelial cells; mEC, microvascular endothelial cells
